# Bacterial Profile and Antibiotic Resistance in Patients with Diabetic Foot Ulcer in Guangzhou, Southern China: Focus on the Differences among Different Wagner's Grades, IDSA/IWGDF Grades, and Ulcer Types

**DOI:** 10.1155/2017/8694903

**Published:** 2017-07-11

**Authors:** Xiaoying Xie, Yunwen Bao, Lijia Ni, Dan Liu, Shaona Niu, Haixiong Lin, Hongyu Li, Chaohui Duan, Li Yan, Songyin Huang, Zhaofan Luo

**Affiliations:** ^1^Department of Clinical Laboratory, Sun Yat-sen Memorial Hospital, Sun Yat-sen University, Guangzhou 510120, China; ^2^Department of Endocrinology, Sun Yat-sen Memorial Hospital, Sun Yat-sen University, Guangzhou 510120, China; ^3^Department of Endocrinology, Linyi People's Hospital, Linyi 276003, China

## Abstract

**Objective:**

To understand the bacterial profile and antibiotic resistance patterns in diabetic foot infection (DFI) in different Wagner's grades, IDSA/IWGDF grades, and different ulcer types in Guangzhou, in order to provide more detailed suggestion to the clinician about the empirical antibiotic choice.

**Methods:**

207 bacteria were collected from 117 DFIs in Sun Yat-sen Memorial Hospital from Jan.1, 2010, to Dec.31, 2015. The clinical data and microbial information were analyzed.

**Results:**

The proportion of Gram-negative bacteria (GNB) was higher than Gram-positive bacteria (GPB) (54.1% versus 45.9%), in which Enterobacteriaceae (73.2%) *and Staphylococcus* (65.2%) were predominant, respectively. With an increasing of Wagner's grades and IDSA/IWGDF grades, the proportion of GNB bacterial infection, especially *Pseudomonas*, was increased. Neuro-ischemic ulcer (N-IFU) was more susceptible to GNB infection. Furthermore, with the aggravation of the wound and infection, the antibiotic resistance rates were obviously increased. GPB isolated in ischemic foot ulcer (IFU) showed more resistance than the N-IFU, while GNB isolates were on the opposite.

**Conclusions:**

Different bacterial profiles and antibiotic sensitivity were found in different DFU grades and types. Clinician should try to stay updated in antibiotic resistance pattern of common pathogens in their area. This paper provided them the detailed information in this region.

## 1. Introduction

Diabetes is a metabolic syndrome characterized by hyperglycemia, which has become a heavy burden to China [1]. Deregulated metabolism in diabetics is link to many complications, including neuropathy, retinopathy, nephropathy, atherosclerosis, and foot ulcers [2]. Diabetic foot ulcer (DFU) is an outcome of complicated amalgam of several risk factors such as peripheral vascular disease, peripheral neuropathy, trauma, and impaired resistance to infection [3], and continues to be a major reason for lower extremity amputation worldwide [4]. Diabetic foot infection (DFI) was considered as one of the most frequent and disastrous complications of diabetes. As reported, 60% of DFU are infected on presentation [4], which can increase the risk of a lower extremity amputation by 50% compared to the DFUs without infection [5, 6]. Because the diabetics' infection can worsen quickly, clinician must pursue the diagnosis aggressively [7] to select an initial antibiotic regimen for the likely pathogens, which need more microbiological information about the DFUs before the wound cultures and antibiotic sensitivity test. Thus, there is an urgent need for the bacterial profile and antibiotic resistance suggestion in more details to give their empirical antibiotic selection “a best guess.”

There were several researches reported that acute DFI is usually caused by aerobic Gram-positive cocci, but deep or chronic wounds often harbor aerobic Gram-negative and obligate anaerobic bacteria, often polymicrobial flora [8–11], while few studies investigated the differences of bacterial profiles in different DFIs in more detail. According to the patients' clinical features acquired at the “first sight” of clinician, including the patient as a whole (e.g., cognitive, metabolic, and fluid status), the affected foot or limb (e.g., the presence of neuropathy and vascular insufficiency) and the infected wound [12], different classification systems are used to assess the severity of DF, the most often used of which were the Wagner-Meggit classification system that takes into consideration the depth of ulcer, presence of gangrene, and level of tissue necrosis [13] and IDSA/IWGDF classification system for defining the presence and severity of an infection of DF [7]. Besides, DF can be classified into three types according to whether with or without peripheral arterial or nerve diseases [9], named ischemic foot ulcer (IFU), neuropathic foot ulcer (NFU), and neuro-ischemic foot ulcer (N-IFU), respectively. More detailed information about pathogens and antibiotic resistance according to different DFU grades and types presents further practical significance for suggesting a more specific antibiotic choice.

On the other hand, to better provide optimal antimicrobial therapy, clinician should be familiar with the common microbial isolates and antibiotic resistance patterns in their own region of practice. Many studies from different regions showed different bacterial profiles in DFIs, especially in warm climate in Asia and Africa [3]. As the main metropolises with a large population and a typical subtropical climate in Southern China, Guangzhou may have a unique bacterial profile and antibiotic resistance in patients with diabetic foot ulcer, while rarely studied.

With the aim of understanding the bacterial profile and antibiotic resistance patterns in DFUs in Guangzhou, furthermore in different Wagner's grades, IDSA/IWGDF grades, and different ulcer types, 117 DFI patients and 207 bacterial isolates were collected from Sun Yat-sen Memorial Hospital from Jan. 1, 2010, to Dec. 31, 2015. The clinical data and microbial information were compared among the different DFUs' grades and types. This knowledge will provide more practical advice about antibiotic agent choice to the clinicians.

## 2. Materials and Methods

### 2.1. Study Design and Patients

A hospital-based retrospect study of 405 inpatients (238 males and 167 females) with DF in the Department of Endocrinology and Metabolism in Sun Yat-sen Memorial Hospital between Jan. 1, 2010, and Dec. 31, 2015, was carried out, including 388 DFU (230 males and 158 females), among which 117 cases presented DFI (72 males and 45 females). Therefore, a total of 117 complete surveys were obtained.

All patients, parents, or guardians signed informed consent approving the use of their specimen samples for research purposes, and the Ethics Committee of Sun Yat-sen Memorial Hospital approved the study. Ethical committee's Reference number: [2017] 伦备第(09)号. Clinical diagnosis of infection was defined by the presence of at least 2 of the following indicators: local swelling or indurations, >0.5 cm of erythema around the wound, local tenderness or pain, local warmth, and purulent discharge [7, 14]. Briefly, clinical severity of ulcer was assessed by Wagner-Meggit classification system [13] and severity of infection was quantified according to the IDSA/IWGDF classification system [7] as previous description. The patients were classified into four Wagner's grades and three IDSA/IWGDF grades based on these systems (Table 1). The diagnosis of peripheral sensory neuropathy was based on failure to appreciate a 10 g Semmes-Weinstein monofilament test and on vibration detection test performed with a 128 Hz tuning fork, and the peripheral arterial disease was diagnosed by limb arterial color Doppler investigation. According to whether with peripheral arterial disease or peripheral sensory neuropathy, the diabetic foot ulcer (DFU) can be classified into ischemic foot ulcer (IFU), neuropathic foot ulcer (NFU), and neuro-ischemic foot ulcer (N-IFU). The DFUs only with peripheral arterial disease were defined as IFU, the IFUs together with peripheral sensory neuropathy were defined as N-IFU, and the DFUs with peripheral sensory neuropathy but without peripheral arterial disease were defined as NFU.

### 2.2. Specimen Collection and Microbiological Culturing

All the specimens were sampled to the microbiology laboratory within 48 h after hospital admission. Swabbing were collected from each wound after the wound had been cleansed (using 0.9% sterile saline and gauze) and debrided (removal of necrotic tissue, foreign material, calluses, and undermined wound edges) [15]. No antimicrobial agent or antiseptic was introduced into the wound before specimen collection. Each wound was swabbed by rotation of a wound swab over a 1 cm^2^ area of the wound for 5 seconds, using sufficient pressure to extract fluid from the inner part of the wound [16]. The specimens were placed into sterile transport containers and sent to the microbiology laboratory for aerobic culturing within 30 minutes. Anaerobic culturing was not performed in this study. Totally, 207 isolates were collected from the 117 patients. To avoid sample duplication, isolates that were consecutively isolated from the same individual were excluded. All isolates underwent phenotypic identification using the VITEK® 2 microbial identification system (bioMérieux, Marcy l'Etoile, France) according to the manufacturer's instructions. Susceptibilities were determined using the disk diffusion method in accordance with the performance standards for antimicrobial susceptibility testing, recommended by the Clinical and Laboratory Standards Institute. *Enterobacter cloacae ATCC 700323* and *Staphylococcus saprophyticus ATCC BAA-750* were used as the quality control strain for phenotypic identification. *Escherichia coli ATCC 25922*, *Pseudomonas aeruginosa ATCC 27853*, and *Staphylococcus aureus ATCC 29213* were used as the quality control strain for antibiotic sensitivity test.

### 2.3. Statistical Analysis

In descriptive statistics, the frequency of isolate distribution and antibiotic resistance was treated as categorical variables. The chi-square or two-sided Fisher's exact test was used to discriminate whether the distributions were significantly different between different groups. The distributed variables were expressed as the mean ± standard deviation and compared by one-way ANOVA. Variables without normal distribution were expressed as the media (interquartile range) and compared by Kruskal-Wallis *H* test. It was considered statistically significant if the two-side *P* value < 0.05. All statistical analyses were carried out using SPSS 19.0 for Windows (IBM). All susceptibility data and molecular test results were analyzed using WHONET software, version 5.6.

## 3. Result

### 3.1. Characteristics of Patients and Wounds

Totally, 95.8% DF patients suffered from DFU (388/405), 30.2% (117/388) of which were clinically infected. Additionally, the DFIs in this study were mainly classified in the moderate or severe grades (Wagner's 2~4 grades and IDSA/IWGDF 2~3 grades), rarely in the mild stage, and only 5 patients were NFU (4.3%). All the patients enrolled were type 2 diabetes ones. The percentage of newly diagnosed DFUs was 26.4%, mainly in Wagner's grades 2 and 4 (32.4% and 37.5%), IDSA/IWGDF grade 2 (38.8%), and N-IFUs (33.8%). With an increasing Wagner's grades and IDSA/IWGDF grades, the serum C-reaction protein (CRP) and procalcitonin (PCT) level had an increased trend (*P* < 0.05). There were no significant differences in the majority of the clinical characteristics examined (Table 1).

### 3.2. Distribution of the Pathogens

**Figure 1:**
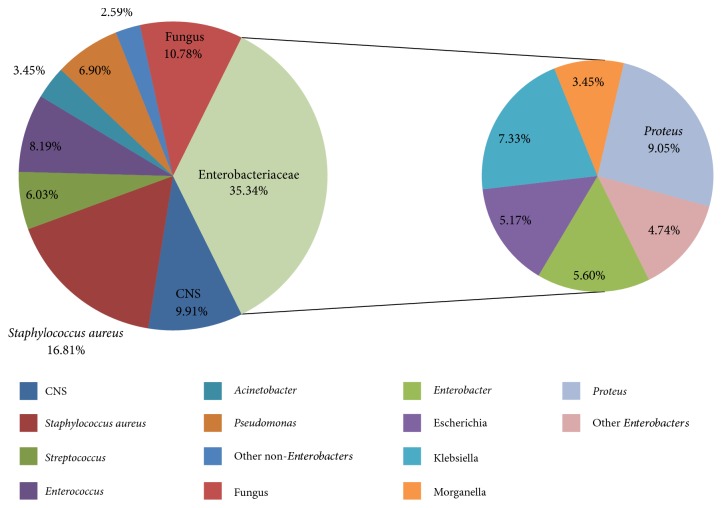
Distribution of the 207 bacteria isolates.

A total of 232 isolates were detected from the 117 specimens, including 207 (89.2%) bacteria and 25 (10.7%) funguses, totally 46 pathogens (Figure 1). In the bacterial infection, the proportion of Gram-negative bacteria (54.1%, 112/207) was higher than Gram-positive bacteria (45.9%, 95/207). Enterobacteriaceae was the main Gram-negative bacteria (73.2%, 82/112), mainly including *Escherichia coli*, *Enterobacter cloacae*, and *Klebsiella pneumonia,* among which the predominant isolates were *Klebsiella pneumonia* (15.2%, 17/112). *Proteus* (18.8%, 21/112) and *Pseudomonas* (14.3%, 16/112) followed*. Staphylococcus* (65.2%, 62/95) is the predominant pathogen in Gram-positive bacteria, main of which was *Staphylococcus aureus* (43.2%, 41/95), followed by *Enterococcus* (20.0%, 19/95). *Candida* was the main pathogen in fungal infection, accounted for 68.0% (17/25) (Figure 1).

**Figure 2:**
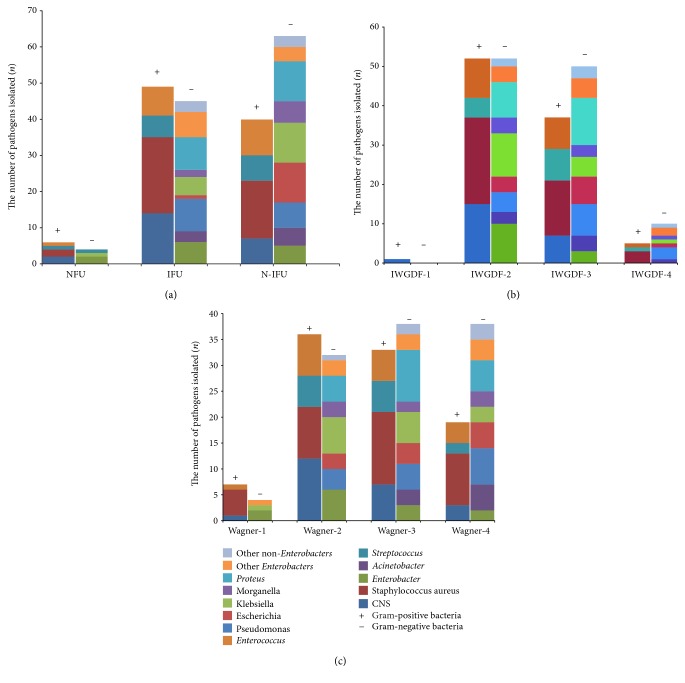
Distribution of bacteria of DFIs of varying Wagner's grades, IDSA/IWGDF grades, and DFU types. (a) The number of bacteria isolated in different DFU types; (b) the number of bacteria isolated in different IDSA/IWGDF grades; (c) the number of bacteria isolated in different Wagner's grades.

With an increasing of Wagner's grades and IDSA/IWGDF grades, the proportion of Gram-negative bacterial infection was obviously increased (Figure 2). *Staphylococcus aureus* and *Enterococcus* were the main Gram-positive bacteria isolated in every Wagner's grades and IWGDF/IDSA grade DFIs, while there was some differences about the Gram-negative isolates in different grade DFIs. Enterobacteriaceae, mainly including *Escherichia coli*, *Enterobacter cloacae*, and *Klebsiella pneumonia,* were the main Gram-negative bacteria isolates in the mild DFIs (Wagner's grade 1 and IWGDF grade 2), and *Proteus* appeared in the moderate wounds (Wagner's grade 2~3 and IWGDF 2~3). Furthermore, *Pseudomonas* and *Acinetobacter* raised to another two main Gram-negative pathogens beyond Enterobacteriaceae in the Wagner's grade 4 ulcers (17.6% and 14.7%, separately), and the proportion of *Pseudomonas* increased in severe infected wound (IWGDF grade 3~4) (27.3%, 3/11), too. Different from IFU and NFU, the N-IFUs were more susceptible to Gram-negative bacterial infection (47.9% and 40.0% versus 61.2%). The bacterial profiles were similar in different DFU types. Details were shown in Table 2 and Figure 2.

More than a half of the DFIs in this study were polymicrobial (59.8%, 70/117), with aerobic Gram-positive cocci (GPC), and especially staphylococci, the most common causative organisms. Especially in the IWGDF grade 2, Wagner's grade 2/4 DFUs, and N-IFU patients (Table 1). All the fungal infections (*n* = 23) were polymicrobial with bacteria.

### 3.3. Antibiotic Resistance and Potential Antibiotics in Different Wounds

MDR (multiple-drug resistance) isolates were broadly distributed in the 207 bacteria isolated from different grades and DFU types (40.5%, 84/207). XDR (extensively drug resistant) isolates accounted for 9.7% in the bacteria (20/207), mainly isolated in Wager's grade 3 (14.1%, 10/71) and IWGEF grade 3 (13.8%, 12/87), especially IFUs (12.8%, 12/94). The definition of MDR and XDR was according to the international expert proposal for interim standard definition for acquired resistance in 2012 [17]. Totally, 22 MRSA were detected in the 207 isolates, distributed in different grades and types. One CRE (carbapenem-resistant Enterobacteriaceae) were isolated from a 49-year-old male patient who was diagnosed DFU four years ago, given systemic and local antibiotic therapy for several times during his three hospitalization periods and outside hospital, whose wound was classified to Wagner grade 3, IWGDF grade 4, and N-IFU when the carbapenem-resistant *Escherichia coli* was isolated. No VRE (vancomycin resistant *Enterococcus*), PDRAB (pandrug-resistant *Acinetobacter baumanii*), and PDRPA (pandrug-resistant *Pseudomonas aeruginosa*) were detected.

As the main pathogens of DFI, the antibiotic sensitivity information of *Staphylococcus aureus* and Enterobacteriaceae was analyzed. Different antibiotic resistance patterns were shown in different wound grades and types.

**Figure 3:**
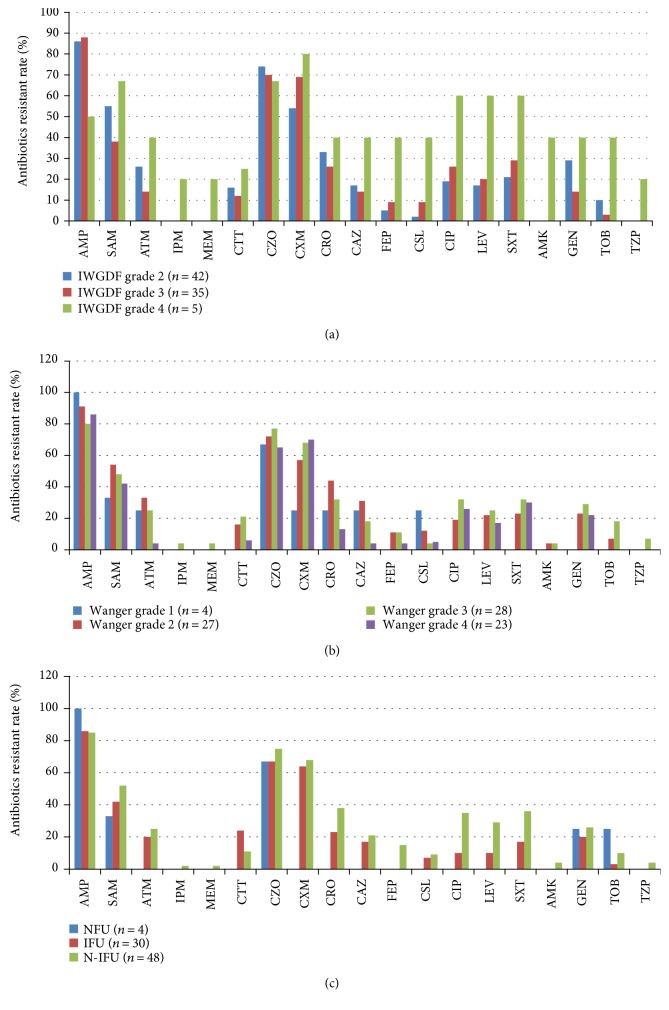
Antibiotic resistance rates of Enterobacteriaceae isolated in DFI in different Wagner's grades, IDSA/IWGDF grades, and DFU types. (a) Antibiotic resistance rates of Enterobacteriaceae isolated in different IDSA/IWGDF grades' DFIs; (b) antibiotic resistance rates of Enterobacteriaceae isolated in different Wagner's grades' DFIs; (c) antibiotic resistance rates of Enterobacteriaceae isolated in different DFU types. AMP, ampicillin; SAM, ampicillin/sulbactam; ATM, aztreonam; IPM, imipenem; MEM, meropenem; CTT, cefotetan; CZO, cephazolin; CXM, cefuroxime; CRO, ceftriaxone; CAZ, ceftazidime; FEP, cefepime; CSL, cefoperazone/sulbactam; CIP, ciprofloxacin; LEV, levofloxacin; SXT, trimethoprim/sulfamethoxazole; AMK, amikacin; GEN, gentamicin; TOB, tobramycin; TZP, piperacillin/tazobactam.

As the representative of Gram-positive cocci, *Staphylococcus aureus* showed a high resistance rate to common antibiotics. High resistance rate to penicillin was detected (92.3%, 36/39), followed by the tetracycline (64.1%, 25/39). However, most of the isolates were susceptible to *β*-L-ase 1(*β*-lactamase inhibitor), including amoxicillin/clavulanate (12.8%, 5/39) and ampicillin/sulbactam (0.0%). All isolates were susceptible to quinupristin-dalfopristin, tigecycline, vancomycin, teicoplanin, and linezolid. With an increasing of Wagner's grades and IDSA/IWGDF grades, the resistance rate to some antibiotics was obviously increased, including penicillin, the third generation cephalosporin (cefatriaxone and ceftazidime), carbapenem (imipenem), fluoroquinolone with good activity against aerobic Gram-positive cocci (levofloxacin, moxifloxacin, and ciprofloxacin), aminoglycosides (gentamycin), erythromycin, rifampicin, and clindamycin (Figures 3(a) and 3(b)). The *Staphylococcus aureus* isolated in IFUs showed more resistant to the antibiotics than the N-IFU, while the NFUs were not discussed due to its rare number (Figure 3(c)).

**Figure 4:**
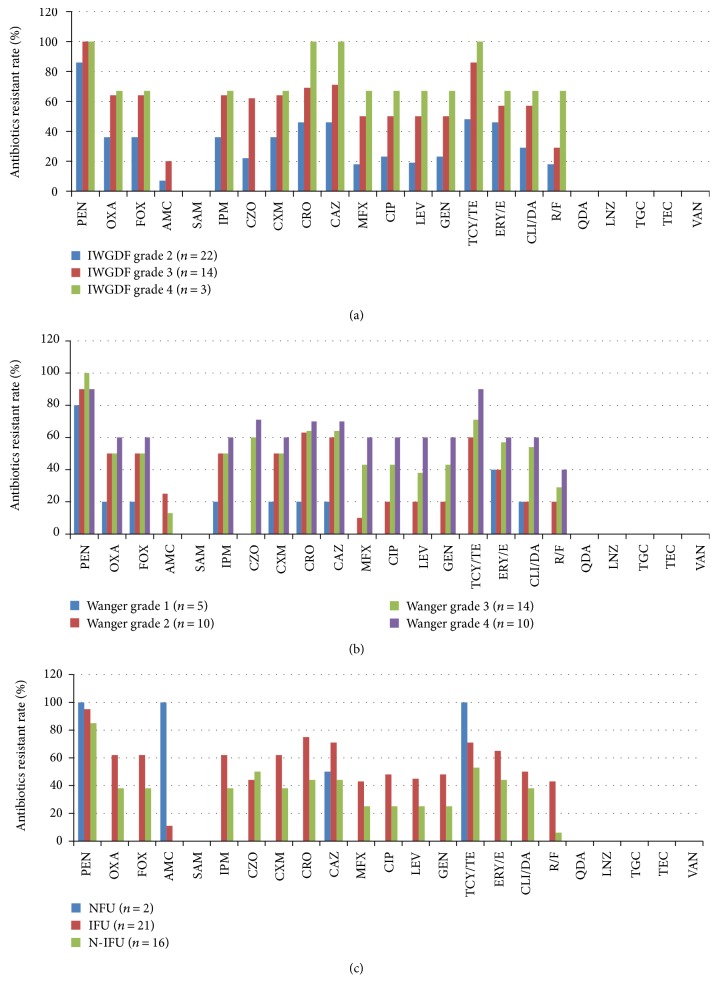
Antibiotic resistance rates of *Staphylococcus aureus* isolated in DFI in different Wagner's grades, IDSA/IWGDF grades, and DFU types. (a) Antibiotic resistance rates of *Staphylococcus aureus* isolated in different IDSA/IWGDF grades' DFIs; (b) antibiotic resistance rates of *Staphylococcus aureus* isolated in different Wagner's grades' DFIs; (c) antibiotic resistance rates of *Staphylococcus aureus* isolated in different DFU types. PEN, penicillin; OXA, oxacillin; FOX, cefoxitin; AMC, amoxicillin/clavulanate; SAM, ampicillin/sulbactam; IPM, imipenem; CZO, cephazolin; CXM, cefuroxime; CRO, ceftriaxone; CAZ, ceftazidime; MFX, moxifloxacin; CIP, ciprofloxacin; LEV, levofloxacin; GEN, gentamicin; TCY/TE, tetracycline; ERY/E, erythromycin; CLI/DA, clindamycin; RIF, rifampicin; QDA, quinupristin/dafoeleptin; LNZ, linezolid; TGC, tigecycline; TEC, teicoplanin; VAN, vancomycin.

Similarly, high resistance rates to the common antibiotics were detected in Enterobacteriaceae. Almost all the isolates were resistant to the ampicillin (85.4%, 70/82), followed by the first/second generation cephalosporin, including cefazolin (72.0%, 59/82) and cefuroxime (64.6%, 53/82), especially in the higher Wagner's grades and IDSA/IWGDF grades. Low resistance rates were detected to carbapenem (1.2%, 1/82), cefoperazone-sulbactam (7.3%, 6/82), the fourth generation cephalosporin (8.5%, 7/82), and tobramycin (8.5%, 7/82). Generally, the resistance to antibiotic increased with the increasing of IDSA/IWGDF grades (Figure 4(a)). While from the aspect of severity of wound, the most serious resistance to antibiotics distributed in Wagner's grade 3, followed by Wagner's grade 2, especially to the cephalosporins (Figure 4(b)). Different from *Staphylococcus aureus*, the Enterobacteriaceae isolates in N-IFU showed more resistant to the antibiotics than the IFU. NFUs were not discussed due to its rare number (Figure 4(c)), as well.

According to the resistance rates of 33 antibiotic agents of the two major pathogens above, we defined the regimens whose resistance rate was <30% as “potential empirical regimens” and the ones whose resistance rate > 70% as “alarming empirical regimens” in every grades and types. Details showed in Table 3.

## 4. Discussion

To our knowledge, this is the first prospective study on microbiological profile and antibiotic resistance pattern of the diabetic foot infection based on the different classification systems, in order to give the clinicians more suggestions in details for initial empirical antibiotic selection according to the comprehensive assessment of the patients.

DFU continues to be a major reason for lower extremity amputation worldwide [4], about half of which are clinically infected at presentation [18]. In our study, 95.8% DF patients suffered from DFU, 30.2% of which were clinically infected, and mainly the chronic ulcer with infection. The polymicrobial infection, including polybacterial infection and bacteria-fungus infection, accounted for 59.8% of the DFIs in this study, which coincide with the previous reports [12, 19]. As the other studies, *Staphylococcus* is the predominant Gram-positive bacteria, including *Staphylococcus aureus* and *CN-S* (*Coagulase-negative staphylococcus*). Compared with the Gram-positive bacteria, there were more species of Gram-negative bacteria infected by DFIs. From the general and species of the bacteria, *Proteus* and *Pseudomonas aeruginosa* were the predominant pathogens in Gram-negative bacteria, followed by *Klebsiella pneumonia*, different from some reports in which the dominating Gram-negative flora was *Escherichia coli* in other areas [20], may due to the warm climates in Guangzhou. However, the predominant flora was Enterobacteriaceae. Coinciding with some studies which showed that the Gram-negative organisms were the most frequent isolates in DFIs in warm climates, especially in Southeast Asia and Africa [21, 22], the prevalence of Gram-negative was some higher than the positive aerobes in this study, as Guangzhou has a warm and humid climates.

To the DFIs, selection of an initial antibiotic regimen is usually empirical, so the likely pathogens and their antibiotic sensitivity often are “guessed” by the clinician before the microorganism cultivation and sensitivity tests. Therefore, a detailed bacterial profile and antibiotic resistance pattern associated with the different severity and types of DFIs is urgently needed for the clinicians. Actually, the severity of the DFU and infection is first determined by the clinical classification scheme. Various classification systems have been proposed to assess the severity of diabetic foot lesion that attempt to encompass different characteristics of ulcer including ulcer size, depth, ischemia, infection, and neuropathy [3]. Wagner-Meggit classification system is the most widely used classification system [23] but cannot help to take into consideration about ischemia and infection. Another classification system given by the Infectious Disease Society of America (IDSA) and International Working Group on the Diabetic Foot (IWGDF) can define the presence and severity of an infection of the diabetic foot, named IWGDF/IDSA classification [7]. Besides, clinician used to classify the DF to IFU (ischemic foot ulcer), NFU (neuropathic foot ulcer), and N-IFU (neuro-ischemic foot ulcer) according to more detailed vessel and nerve check.

In this study, different bacterial profiles and antibiotic sensitivity were found in different Wagner's grade, IWGDF grade, and DFU types. With the aggravation of the wound and infection, the Gram-negative bacterial species harbored and increased, especially the proportion of *Pseudomonas*, a common nosocomial infection bacteria, resistant to many kinds of antibiotics, which coincided with the previous study [24]. The polymicrobial infection distributed mainly in moderate wound (IWGDF grade 2 and Wagner's grade 2 DFUs) and N-IFU patients, which was beyond our expectation that the severe wound and infection may tend more polymicrobial. Combined with the clinical characteristics, the patients in moderate wound and N-IFUs in our study had more newly diagnosed rate compared with the other groups, who had not received systemic antibiotic treatments, which may cause the results above.

Selection of an initial antibiotic regimen is usually empirical, that is, the best guess at what agents will cover the likely pathogens. In details, this study gave the clinician suggestions about the most possible regimens as the “potential antibiotics,” and the regimens should not be used for their high resistance as the “alarming antibiotics” according to different wounds. When the patients were evaluated by Wagner-Meggit classification system and IWGDF/IDSA classification system, and the ulcers were typed as IFU, NFU, or N-IFU, clinicians can choose the overlapping of different systems according to Table 3. For example, if the wound of a DFI patient was graded as Wagner-Meggit grade 2 and IWGDF/IDSA grade 3 and was diagnosed as an ischemic foot ulcer (IFU), combined with the bacterial profile and antibiotic resistance, the clinician can try cefotetan, *β*-L-ase, carbapenem, fluoroquinolone, or aminoglycosides as the empirical antibiotics to cover the main possible pathogens and avoid penicillin, ampicillin, the first to third generation cephalosporin and tetracycline in order to prevent the infective treatment and MDR bacteria due to antibiotic abuse. If the DFU patient was classified in more severe IWGDF grades, less potential empirical regimens could be chosen and more should be avoided, then modified the regimens according to the available clinical and microbiological information.

However, this paper only provided the empirical regimens selected suggestion about the predominant GNB and GPB, while did not cover all the pathogens. Actually, some other pathogens showed higher resistance rates to more antibiotics due to their natural resistance, for example, the *Pseudomonas aeruginosa*, *Enterococcus faecium*, and *Stenotrophomonas maltophilia*. Therefore, more attention should be paid to the DFIs with high risk of the natural resistance pathogens above, like the Wagner's grade 4 and IWGDF/IDSA grade 4 wound.

The major limitation of this study is the lack of anaerobic culturing. Further study is required to evaluate the anaerobic distribution and drug sensitivity in the different grades of DFUs. Another limitation is the small number of included patients, especially those with Wagner-Meggit grade 1 or IWGDF/IDSA grade 1 wound, and rarely neuropathic ulcerations. Tissue biopsy is known as the most standard method, and swab cultures are considered as not reliable since it generally includes the colonizers and not the causative pathogen [15]. But in this study, the swabs were obtained after a complete debridement in order to avoid the colonizers, and the CNS, as the main colonized organisms in the skin, were detected lower than 10% in this study, which showed that the swabs were reliable.

## 5. Conclusions

Different bacterial profiles and antibiotic sensitivity were found in different Wagner's grades, IWGDF grades, and DFU types. Clinician should try to stay updated in antibiotic resistance pattern of common pathogens in their area, especially when practice on the empirical antibiotic use. This paper provided the detailed practical information (potential empirical regimens and alarming empirical regimens) to the clinician based on the assessments to the DFIs from the different aspects in this region.

## Figures and Tables

**Table 1:** Characteristics of DFIs of varying Wagner's grades, IDSA/IWGDF grades, and DFU types.

Clinical characteristic	Wagner's grades				IDSA/IWGDF grades				DFU types		
	1	2	3	4	1	2	3	4	IFU	NFU	N-IFU
Number of patients (*n*)	7	34	44	32	1	54	53	9	53	5	59
Mean age (years, y)^a^	66.7 ± 14.8	64.7 ± 9.7	64.0 ± 11.0	66.1 ± 11.6	63.4	65.9 ± 11.2	65.0 ± 11.6	62.2 ± 11.5	64.8 ± 11.5	63.4 ± 14.0	65.5 ± 11.0
Male/female (*n*)	2/5	20/14	30/14	20/12	1/0	34/20	32/21	5/4	35/18	2/3	35/24
Duration of diabetes	1.5 (59.0)	2.5 (23.0)	18.6 (24.5)	4.2 (7.6)	1.8	2.7 (23.9)	6.5 (23.5)	2.8 (13.6)	4 (23.0)	24 (31.3)	6.8 (10.5)
(months, m)^b^											
BMI^a^	24.5 ± 6.0	24.5 ± 5.6	23.4 ± 2.7	27.0 ± 2.1	25.9	24.0 ± 4.4	27.7 ± 2.1	23.3 ± 2.2	28.3 ± 2.1	24.9 ± 3.9	23.4 ± 3.8
HbA1c (%)^a^	6.5 ± 1.4	8.9 ± 2.0	8.0 ± 2.3	8.8 ± 2.9	7.9	8.6 ± 2.4	7.9 ± 2.1	9.9 ± 3.2	8.6 ± 2.2	9.6 ± 2.8	8.3 ± 2.6
CRP (mg/L)^b^^∗^	19.7 (76.3)	22 (46.5)	25.5 (72.8)	91.9 (226.9)	11	21.8 (47.5)	49.3 (117.9)	197.6 (231.4)	22.7 (69.5)	10.9 (129.0)	70 (82.7)
PCT (mmol/L)^b^^∗^	0.2 (0.1)	0.2 (0.3)	1.0 (0.3)	2.5 (0.6)	0	0.2 (0.2)	0.7 (0.1)	2.4 (0.4)	0.2 (0.1)	0.1 (0.0)	0.7 (0.2)
Newly diagnosed^c^^∗^	1 (14.3)	11 (32.4)	7 (15.9)	12 (37.5)	0 (0.0)	21 (38.8)	8 (15.1)	2 (22.2)	11 (20.75)	0 (0.0)	20 (33.8)
Polymicrobial infection^c^	4 (57.1)	22 (64.7%)	24 (54.5%)	20 (62.5%)	0 (0.0)	38 (70.4%)	27 (50.9%)	6 (66.7%)	28 (52.8%)	1 (20.0%)	41 (69.5%)

^a^Expressed as mean ± SD; ^b^expressed as media (interquartile range); ^c^expressed as number (percentage); BMI: body mass index; CRP: C-reaction protein; PCT: procalcitonin. ^∗^*P* < 0.05, compared between different groups.

**Table 2:** Distribution of bacteria of DFIs of varying Wagner's grades, IDSA/IWGDF grades, and DFU types.

Classification (*n*)^a^	Gram-positive coccus *n* (%)				Gram-negative bacilli *n* (%)			
	*Staphylococcus aureus*	CNS	*Streptococcus*	*Enterococcus*	Enterobacteriaceae	*Acinetobacter*	*Pseudomonas aeruginosa*	Other non-*Enterobacters*
DFU type								
NFU (10)	2 (20.0)	2 (20.0)	1 (10.0)	1 (10.0)	4 (40.0)	0	0	0
IFU (94)	21 (22.3)	14 (14.9)	6 (6.4)	8 (8.5)	30 (31.9)	3 (3.0)	9 (9.6)	3 (3.2)
N-IFU (103)	16 (15.5)	7 (6.8)	7 (6.8)	10 (9.7)	48 (46.6)	5 (4.9)	7 (6.8)	3 (2.9)
Wagner's grades								
Grade 1 (11)	5 (45.5)	1 (9.1)	0	1 (9.1)	4 (36.4)	0	0	0
Grade 2 (68)	10 (14.7)	12 (17.6)	6 (8.8)	8 (11.7)	27 (39.4)	0	4 (5.9)	1 (1.4)
Grade 3 (71)	14 (19.7)	7 (9.8)	6 (8.5)	6 (8.5)	28 (39.4)	3 (4.2)	5 (7.0)	2 (2.8)
Grade 4 (57)	10 (17.5)	3 (5.3)	2 (3.5)	4 (7.0)	23 (40.3)	5 (8.7)	7 (12.3)	3 (5.3)
IWGDF grades								
Grade 1 (1)	0	1 (100.0)	0	0	0	0	0	0
Grade 2 (104)	22 (21.2)	15 (14.4)	5 (4.8)	10 (9.6)	42 (40.4)	3 (2.8)	5 (4.8)	2 (1.9)
Grade 3 (87)	14 (16.1)	7 (8.0)	8 (9.2)	8 (9.2)	35 (40.2)	4 (4.6)	8 (9.2)	3 (3.4)
Grade 4 (15)	3 (20.0)	0	1 (6.7)	1 (6.7)	5 (33.3)	1 (6.7)	3 (20.0)	1 (6.7da)

^a^The numbers of the pathogens isolated; CNS: coagulase negative *staphylococcus*, mainly including *Staphylococcus epidermidis* and *Staphylococcus haemolyticus*; *Streptococcus*, including *Streptococcus agalactiae* and Group G *Streptococcus*; Enterobacteriaceae, mainly including *Escherichia coli*, *Enterobacter cloacae*, *Klebsiella pneumonia*, and *Proteus*; *Acinetobacter*, mainly *Acinetobacter baumanii*; other non-*Enterobacters*, mainly including *Enterobacter cloacae* and *Stenotrophomonasmaltophilia.*

**Table 3:** Potential and alarming empirical regimens for different Wagner's grades, IDSA/IWGDF grades, and types of diabetic foot infections.

Classification	Usual pathogen(s)^a^	Potential empirical regimens^b^	Alarming empirical regimens^c^
Type			
NFU	Enterobacteriaceae	Aztreonam; cefotetan; 2nd~4th gen ceph; carbapenem; FQ; cipro; T/S; *β*-L-ase 2; aminoglycosides	First gen ceph; ampicillin
IFU	Enterobacteriaceae	Aztreonam; 3rd/4th gen ceph; carbapenem; FQ; cipro; T/S; *β*-L-ase 2; aminoglycosides	Ampicillin
	*Staphylococcus aureus*	*β*-L-ase 1	Pen; second/third gen ceph; tetracycline
N-IFU	Enterobacteriaceae	Aztreonam; cefotetan; 3rd/4th gen ceph; carbapenem; *β*-L-ase 2; aminoglycosides	First/second gen ceph; ampicillin
	*Staphylococcus aureus*	*β*-L-ase 1; aminoglycosides; quinolones; rifampin; FQ; cipro	Pen
Wagner's grade			
1~2	*Staphylococcus aureus*	*β*-L-ase 1; aminoglycosides; rifampin; FQ; cipro	Pen
	Enterobacteriaceae	Aztreonam; cefotetan; 3rd/4th gen ceph; carbapenem; FQ; cipro; T/S; *β*-L-ase 2; aminoglycosides	First gen ceph; ampicillin
3	Enterobacteriaceae	Aztreonam; cefotetan; 3rd/4th gen ceph; carbapenem; FQ; *β*-L-ase 2; aminoglycosides	First/second gen ceph; ampicillin
	*Staphylococcus aureus*	*β*-L-ase 1	Pen; tetracycline
4	Enterobacteriaceae	Same to Wanger grade 3	First/second gen ceph; ampicillin
	*Staphylococcus aureus*	Same to Wanger grade 3	Pen; first~third gen ceph; tetracycline
IWGDF grade			
1~2	Enterobacteriaceae	Aztreonam; cefotetan; 2nd~4th gen ceph; carbapenem; FQ; cipro; clindamycin; T/S; *β*-L-ase 2; aminoglycosides	First gen ceph; ampicillin
	*Staphylococcus aureus*	*β*-L-ase 1; aminoglycosides; rifampin; FQ; cipro;	Pen
3	Enterobacteriaceae	aztreonam; cefotetan; 2nd ~ 4th gen ceph; carbapenem; FQ; cipro; *β*-L-ase 2; aminoglycosides	First/second gen ceph; ampicillin
	*Staphylococcus aureus*	*β*-L-ase 1; rifampin	Pen; third gen ceph; tetracycline
4	Enterobacteriaceae	Carbapenem; *β*-L-ase 2	First/second gen ceph; ampicillin
	*Staphylococcus aureus*	*β*-L-ase 1	Pen; first~third gen ceph; tetracycline

*β*-L-ase: *β*-lactam, *β*-lactamase inhibitor; *β*-L-ase 1: amoxicillin/clavulanate, ampicillin/sulbactam; *β*-L-ase 2: ticarcillin/clavulanate, piperacillin/tazobactam; group 1: carbapenem, ertapenem; group 2: carbapenem, imipenem, meropenem; ceph: cephalosporin; gen: generation; FQ: fluoroquinolone with good activity against aerobic Gram-positive cocci (e.g., levofloxacin or moxifloxacin); cipro: antipseudomonal fluoroquinolone, for example, ciprofloxacin; T/S; trimethoprim/sulfamethoxazole; pen: penicilin. ^a^All the *Staphylococcus aureus* isolates were sensetive to teicoplanin, linezolid, tigecycline, and vancomycin; ^b^the agents whose resistant rates were <30%; ^c^the agents whose resistant rates were >70%.
